# The Effect of the Intraoperative Blood Loss and Intraoperative Blood Transfusion on the Short-Term Outcomes and Prognosis of Colorectal Cancer: A Propensity Score Matching Analysis

**DOI:** 10.3389/fsurg.2022.837545

**Published:** 2022-04-04

**Authors:** Bing Kang, Xiao-Yu Liu, Zi-Wei Li, Chao Yuan, Bin Zhang, Zheng-Qiang Wei, Dong Peng

**Affiliations:** ^1^Department of Clinical Nutrition, The First Affiliated Hospital of Chongqing Medical University, Chongqing, China; ^2^Department of Gastrointestinal Surgery, The First Affiliated Hospital of Chongqing Medical University, Chongqing, China

**Keywords:** colorectal cancer, intraoperative blood loss, surgery, prognosis, intraoperative blood transfusion, outcomes

## Abstract

**Purpose:**

The purpose of the current study was to analyze the effect of intraoperative blood loss (IBL) and intraoperative blood transfusion (IBT) on the short-term outcomes and prognosis for patients who underwent primary colorectal cancer (CRC) surgery.

**Methods:**

We retrospectively collected the patients' information from the database of a teaching hospital from January 2011 to January 2020. IBL and IBT were collected and analyzed, and the propensity score matching (PSM) analysis was performed.

**Results:**

A total of 4,250 patients with CRC were included in this study. There were 1,911 patients in the larger IBL group and 2,339 patients in the smaller IBL group. As for IBT, there were 82 patients in the IBT group and 4,168 patients in the non-IBT group. After 1:1 ratio PSM, there were 82 patients in the IBT group and 82 patients in the non-IBT group. The larger IBL group had longer operation time (*p* = 0.000 < 0.01), longer post-operative hospital stay (*p* = 0.000 < 0.01), smaller retrieved lymph nodes (*p* = 0.000 < 0.01), and higher overall complication (*p* = 0.000 < 0.01) than the smaller IBL group. The IBT group had longer operation time (*p* = 0.000 < 0.01), longer hospital stay (*p* = 0.016 < 0.05), and higher overall complications (*p* = 0.013 < 0.05) compared with the non-IBT group in terms of short-term outcomes. Larger IBL (*p* = 0.000, HR = 1.352, 95% CI = 1.142–1.601) and IBT (*p* = 0.044, HR = 1.487, 95% CI = 1.011–2.188) were independent predictive factors of overall survival (OS). Larger IBL (*p* = 0.000, HR = 1.338, 95% CI = 1.150–1.558) was an independent predictor of disease-free survival (DFS); however, IBT (*p* = 0.179, HR = 1.300, 95% CI = 0.886–1.908) was not an independent predictor of DFS.

**Conclusion:**

Based on the short-term outcomes and prognosis of IBL and IBT, surgeons should be cautious during the operation and more careful and proficient surgical skills are required for surgeons.

## Introduction

Colorectal cancer (CRC) is one of the most common cancers in the world, with an estimated 1.8 million new cases and 861,000 deaths each year (1). The incidence of CRC is decreasing in western countries including the United States, Canada, and Australia. However, an upward trend has been existing in China; moreover, China is a country with the largest number of new cases and deaths of CRC every year in the world (2). At present, radical surgical treatment of CRC is still the most important and decisive treatment (3–5).

Due to the popularity of laparoscopic surgery and the development of surgical equipment such as electrothermal bipolar activation devices and ultrasound systems, the average intraoperative blood loss (IBL) has decreased (6, 7). However, IBL is still a matter of concern during the operation. Studies have found that larger IBL might affect complications and prognosis in gastrointestinal tumors (8–10).

Perioperative blood transfusion is another concern for surgeons. However, the influence of perioperative blood transfusion on short-term outcomes and prognosis was inconsistent. Some studies reported that perioperative blood transfusion could increase post-operative complications, prolong hospital stay, and affect prognosis (11–13). However, other studies suggested that perioperative blood transfusion did not affect prognosis (14, 15).

The effect of IBL in patients with CRC is still controversial. Some studies reported that larger IBL could increase complications and reduce prognosis (16–18). However, other studies reported that IBL had no effect on complications and prognosis (19, 20). Furthermore, no previous studies reported the intraoperative blood transfusion (IBT) on the outcomes and prognosis of patients with CRC. Thus, the purpose of the current study was to analyze the effect of IBL and IBT on the short-term outcomes and prognosis of patients who underwent primary CRC surgery.

## Materials and Methods

We retrospectively collected the patients' information from database of a teaching hospital. The database included patients who underwent primary CRC surgery from Jan 2011 to Jan 2020. This study was conducted in accordance with the World Medical Association Declaration of Helsinki. Ethical approval from the institutional review board of the First Affiliated Hospital of Chongqing Medical University was obtained (2021-540). All patients signed the informed consent.

### Patients Selection

**Figure 1:**
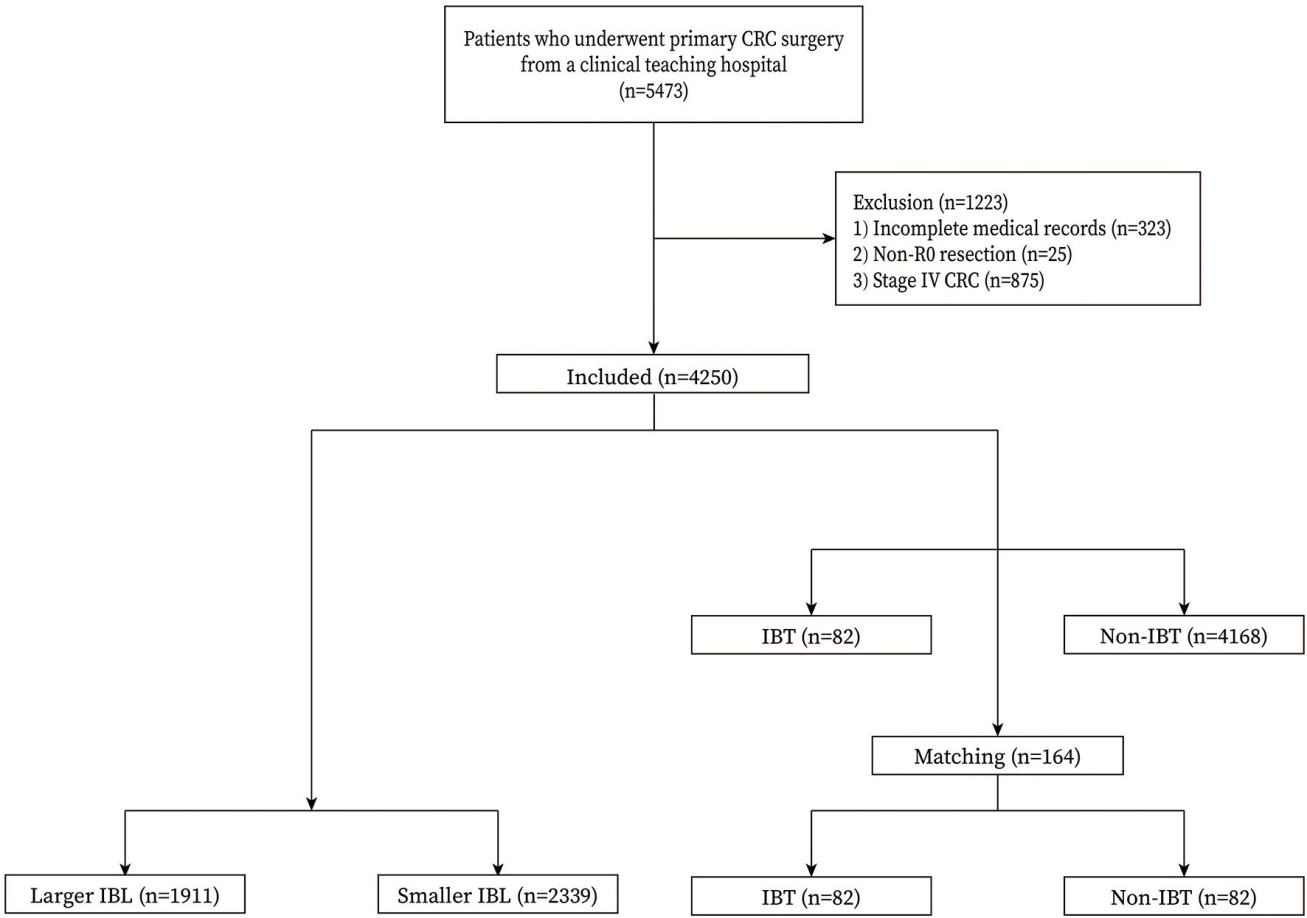
Flow chart of patient selection. IBL, intraoperative blood loss; IBT, intraoperative blood transfusion.

We included patients who underwent primary CRC surgery in a single teaching hospital (*n* = 5,473). The exclusion criteria were as follows: 1, incomplete medical records (*n* = 323); 2, non-R0 resection (*n* = 25); and 3, stage IV CRC (*n* = 875). Finally, a total of 4,250 patients with CRC were included in this study (Figure 1).

### Surgery Management

The CRC surgery was conducted according to the principle of AJCC 8th Edition (16). Radical resection (total mesorectal excision/complete mesocolic excision) was performed, and the pathology confirmed R0 resection.

### Definitions

The tumor node metastasis stage was diagnosed according to the AJCC 8th Edition (21). The complications were graded according to the Clavien-Dindo classification (22), and major complications were defined as ≥ III classification complications. Overall survival (OS) was defined as the time from surgery to the last follow-up or death. Disease-free survival (DFS) was defined as the time from surgery to recurrence, death, or last follow-up. IBL was defined as estimated blood loss during CRC surgery, and IBL was divided into two groups including the larger IBL and the smaller IBL group. The cut-off value of IBL was the 75th percentile of IBL (100 ml), larger IBL group was defined as IBL ≥ 100 ml, smaller IBL group was defined as IBL < 100 ml. IBT was defined as patients who underwent blood transfusion during CRC surgery, and they were divided into two groups including IBT group and non-IBT group.

### Outcomes

The primary outcome was the prognosis including OS and DFS. The second outcome was the short-term outcomes including operation time, retrieved lymph nodes, overall complications, major complications, and post-operative hospital stay.

### Data Collection

The baseline information of patients with CRC was collected retrospectively including sex, age, body mass index (BMI), smoking, drinking, hypertension, type 2 diabetes mellitus (T2DM), tumor location, surgery history, surgical methods (open surgery or laparoscopic surgery), and tumor stage. The short-term outcomes were collected through inpatient medical system. The follow-up information was collected through out-patient system and telephone interview.

### Propensity Score Matching

Propensity score matching (PSM) is a method that could minimize the bias of baseline information (23, 24). In this study, we compared the short-term outcomes and prognosis of IBT on patients with CRC using PSM. Nearest neighbor matching was performed without replacement at a 1:1 ratio and a caliper width with a 0.01 standard deviation was specified. The matched baseline information was as follows: age, sex, BMI, smoking, drinking, hypertension, T2DM, tumor location, and tumor stage.

### Statistical Analysis

Continuous variables are expressed as the mean ± standard deviation (SD) and independent-sample *t*-test was analyzed. Frequency variables are expressed as *n* (%) and Chi-square test or Fisher's exact test was used. Pearson's correlation coefficients were used to analyze the correlation between IBL and clinical characteristics (age, BMI, retrieved lymph nodes, operation time, and post-operative hospital stay). The Kaplan–Meier curve was conducted to compare the difference between the larger IBL group and the smaller IBL group, and between the IBT group and the non-IBT group. Cox regression analyses were performed to identify independent predictive factors for OS and DFS. Data were analyzed using SPSS (version 22.0) statistical software. A bilateral *p* < 0.05 was considered statistically significant.

## Results

### Patients

A total of 4,250 patients with CRC were included in this study. There were 1,911 patients in the larger IBL group and 2,339 patients in the smaller IBL group. As for IBT, there were 82 patients in the IBT group and 4,168 patients in the non-IBT group. After 1:1 ratio PSM, there were 82 patients in the IBT group and 82 patients in the non-IBT group. The inclusion and exclusion criteria, and patients with CRC before and after PSM are shown in Figure 1.

### Clinical Characteristics of Patients With CRC

The clinical information and surgery outcomes are summarized in Table 1. There were 2,496 (58.7%) men and 1,754 (41.3%) women. The average IBL was 100.4 ± 125.7 ml and 82 (1.9%) patients underwent IBT.

**Table 1:** Clinical characteristics of CRC patients.

**Characteristics**	**No. 4250**
Age (mean ± SD), year	62.9 ± 12.1
Sex
Male	2,496 (58.7%)
Female	1,754 (41.3%)
BMI (mean ± SD), kg/m^2^	22.7 ± 3.2
Smoking	1,607 (37.8%)
Drinking	1,301 (30.6%)
Hypertension	1,108 (26.1%)
T2DM	521 (12.3%)
Surgery history	995 (23.4%)
Laparoscopic surgery	3,608 (84.9%)
Tumor location	
Colon	2,308 (54.3%)
Rectum	1,942 (45.7%)
TNM stage	
I	850 (20.0%)
II	1,832 (43.1%)
III	1,568 (36.9%)
IBL, ml	100.4 ± 125.7
IBT	82 (1.9%)
Retrieved lymph nodes	14.7 ± 7.5
Operation time, min	222.8 ± 76.3
Post-operative hospital stay, day	11.4 ± 8.8
Overall complications	927 (21.8%)
Major complications	99 (2.3%)

Variables are expressed as the mean ± SD, n (%), *P < 0.05.

*T2DM, type 2 diabetes mellitus; BMI, body mass index; IBL, intraoperative blood loss; IBT, intraoperative blood transfusion*.

### Baseline Characteristics of IBL

There were 1,911 patients in the larger IBL group and 2,339 patients in the smaller IBL group. The larger IBL group had more men (*p* = 0.000 < 0.01), higher smoking (*p* = 0.000 < 0.01), higher drinking (*p* = 0.038 < 0.05), and more open surgery (*p* = 0.004 < 0.01) than the smaller IBL group (Table 2).

**Table 2:** Baseline characteristics of between larger IBL group and smaller IBL group.

**Characteristics**	**Larger IBL (1,911)**	**Smaller IBL (2,339)**	***P*-value**
Age (year)	62.6 ± 11.9	63.9 ± 12.5	0.135
Sex			0.000*
Male	1,200 (62.8%)	1,296 (55.4%)	
Female	711 (37.2%)	1,043 (44.6%)	
BMI (kg/m^2^)	22.8 ± 3.3	22.6 ± 3.2	0.116
T2DM	283 (14.8%)	238 (10.2%)	0.726
Smoking	789 (41.3%)	818 (35.0%)	0.000*
Drinking	616 (32.2%)	685 (29.3%)	0.038*
Hypertension	488 (25.5%)	620 (26.5%)	0.473
Surgery history	468 (24.5%)	527 (22.5%)	0.134
Open surgery	479 (25.1%)	163 (7.0%)	0.000*
Tumor location			0.092
Colon	855 (44.7%)	1,107 (47.3%)	
Rectum	1,056 (55.3%)	1,232 (52.7%)	
Tumor stage			0.133
I	362 (18.9%)	488 (20.9%)	
II	853 (44.6%)	979 (41.9%)	
III	696 (36.5%)	872 (37.2%)	

Variables are expressed as the mean ± SD, n (%), *P < 0.05.

*T2DM, type 2 diabetes mellitus; BMI, body mass index; IBL, intraoperative blood loss*.

### Short-Term Outcomes of IBL

The short-term outcomes were calculated between the larger IBL group and the smaller IBL group. In this study, the larger IBL group had a longer operation time (*p* = 0.000 < 0.01), longer post-operative hospital stay (*p* = 0.000 < 0.01), smaller retrieved lymph nodes (*p* = 0.000 < 0.01), and higher overall complication (*p* = 0.000 < 0.01) than the smaller IBL group (Table 3).

**Table 3:** Outcomes between larger IBL group and smaller IBL group.

**Characteristics**	**Larger IBL (1,911)**	**Smaller IBL (2,339)**	***P*-value**
Operation time (min)	256.1 ± 80.9	196.2 ± 60.5	0.000*
Retrieved lymph nodes	13.5 ± 7.4	15.7 ± 7.5	0.000*
Post-operative hospital	13.3 ± 10.4	9.8 ± 6.8	0.000*
stay (day)			
Overall complications	538 (28.2%)	389 (16.6%)	0.000*
Major complications	53 (2.8%)	46 (2.0%)	0.083

Variables are expressed as the mean ± SD, n (%), *P < 0.05.

### Correlation of IBL and Clinical Characteristics

Correlation of IBL and clinical characteristics were analyzed. Retrieved lymph nodes (*r* = −0.110, *p* = 0.000 < 0.01), operation time (*r* = 0.369, *p* = 0.000 < 0.01), and post-operative stay (*r* = 0.188, *p* = 0.000 < 0.01) were significantly correlated with IBL (Table 4).

**Table 4:** Correlation of IBL and clinical characteristics.

	**IBL, ml**	
	**Correlation**	***P***
Age, year	0.020	0.191
BMI, kg/m^2^	0.023	0.132
Retrieved lymph nodes	−0.110	0.000*
Operation time, min	0.369	0.000*
Post-operative hospital stay, day	0.188	0.000*

*P < 0.05.

*BMI, body mass index; IBL, intraoperative blood loss*.

### Baseline Characteristics of IBT

The baseline characteristics were compared between the IBT group and the non-IBT group. The IBT group had older age (*p* = 0.004 < 0.01), lower BMI (*p* = 0.001 < 0.01), lower portion of rectal cancer (*p* = 0.003 < 0.01), and lower portion of stage I CRC (*p* = 0.013 < 0.05) than the non-IBT group before PSM. After 1:1 ratio PSM, there was no significant difference between the two groups (*p* > 0.05) (Table 5).

**Table 5:** Baseline characteristics before and after PSM.

**Characteristics**	**Before PSM**			**After PSM**		
	**IBT (82)**	**Non-IBT (4,168)**	***P*-value**	**IBT (82)**	**Non-IBT (82)**	***P*-value**
Age (year)	66.8 ± 14.9	62.8 ± 12.1	0.004*	66.8 ± 14.9	65.3 ± 10.5	0.463
Sex			0.625			0.875
Male	46 (56.1%)	2,450 (58.8%)		46 (56.1%)	47 (57.3%)	
Female	36 (43.9%)	1,718 (41.2%)		36 (43.9%)	35 (42.7%)	
BMI (kg/m^2^)	21.6 ± 3.1	22.8 ± 3.2	0.001*	21.6 ± 3.1	21.6 ± 3.2	0.986
Smoking	29 (35.4%)	1,578 (37.9%)	0.645	29 (35.4%)	31 (37.8%)	0.746
Drinking	23 (28.0%)	1,278 (30.7%)	0.611	23 (28.0%)	23 (28.0%)	1.000
Hypertension	20 (24.4%)	1,088 (26.1%)	0.726	20 (24.4%)	16 (19.5%)	0.450
T2DM	14 (17.1%)	507 (12.2%)	0.179	14 (17.1%)	15 (18.3%)	0.838
Tumor location			0.003*			0.268
Colon	51 (62.2%)	2,257 (54.2%)		51 (62.2%)	44 (53.7%)	
Rectum	31 (37.8%)	1,911 (45.8%)		31 (37.8%)	38 (46.3%)	
Tumor stage			0.013*			0.618
I	7 (8.5%)	843 (20.2%)		7 (8.5%)	10 (12.2%)	
II	35 (42.7%)	1,797 (43.1%)		35 (42.7%)	30 (36.6%)	
III	40 (48.8%)	1,528 (36.7%)		40 (48.8%)	42 (51.2%)	

Variables are expressed as the mean ± SD, n (%), *P < 0.05.

*T2DM, type 2 diabetes mellitus; BMI, body mass index; PSM, propensity score matching; IBT, intraoperative blood transfusion*.

### Short-Term Outcomes of IBT

The IBL of the IBT group was 376.6 ± 327.2 ml, which was larger than 95.0 ± 111.8 ml of the non-IBT group before PSM. After 1:1 ratio PSM, the IBL of the IBT group was 376.6 ± 327.2 ml, which was larger than 86.8 ± 74.7 ml of the non-IBT group.

Before 1:1 ratio PSM, the IBT group had longer operation time (*p* = 0.000 < 0.01), longer hospital stay (*p* = 0.000 < 0.01), and higher overall complications (*p* = 0.000 < 0.01) compared with the non-IBT group.

After 1:1 ratio PSM, the IBT group had longer operation time (*p* = 0.000 < 0.01), longer hospital stay (*p* = 0.016 < 0.05), and higher overall complications (*p* = 0.013 < 0.05) compared with the non-IBT group (Table 6).

**Table 6:** Short-term outcomes before and after PSM.

**Characteristics**	**Before PSM**			**After PSM**		
	**IBT (82)**	**Non-IBT (4,168)**	***P*-value**	**IBT (82)**	**Non-IBT (82)**	***P*-value**
IBL (ml)	376.6 ± 327.2	95.0 ± 111.8	0.000*	376.6 ± 327.2	86.8 ± 74.7	0.000*
Operation time (min)	279.5 ± 91.7	221.7 ± 75.5	0.000*	279.5 ± 91.7	212.5 ± 72.7	0.000*
Retrieved lymph nodes	14.0 ± 7.3	14.7 ± 7.5	0.375	14.0 ± 7.3	15.3 ± 6.6	0.238
Post-operative hospital stay (day)	15.2 ± 9.2	11.3 ± 8.8	0.000*	15.2 ± 9.2	11.7 ± 9.3	0.016*
Overall complications	35 (42.7%)	892 (21.4%)	0.000*	35 (42.7%)	20 (24.4%)	0.013*
Major complications	2 (2.4%)	97 (2.3%)	0.717	2 (2.4%)	3 (3.7%)	1.000

Variables are expressed as the mean ± SD, n (%), *P < 0.05.

*PSM, propensity score matching; IBT, intraoperative blood transfusion*.

### Prognosis

The median follow-up time was 37 (1–114) months. We conducted univariate and multivariate analysis of OS; older age (*p* = 0.000, HR = 1.947, 95% CI = 1.648–2.302), advanced tumor stage (*p* = 0.000, HR = 2.098, 95% CI=1.853–2.377), larger IBL (*p* = 0.000, HR = 1.352, 95% CI = 1.142–1.601), IBT (*p* = 0.044, HR = 1.487, 95% CI = 1.011–2.188), overall complications (*p* = 0.000, HR = 1.439, 95% CI = 1.201–1.723) and major complications (*p* = 0.000, HR = 2.431, 95% CI = 1.708–3.459) were independent predictors of OS (Table 7).

**Table 7:** Univariate and multivariate analysis of overall survival.

**Risk factors**	**Univariate analysis**		**Multivariate analysis**	
	**HR (95% CI)**	***P*-value**	**HR (95% CI)**	***P*-value**
Age (>/ ≤ 64, years)	2.141 (1.817–2.522)	0.000*	1.947 (1.648–2.302)	0.000*
Sex (female/male)	0.897 (0.763–1.055)	0.188		
BMI (>/ ≤ 22.6)	0.793 (0.675–0.930)	0.004*	0.873 (0.743–1.027)	0.101
Hypertension (yes/no)	1.047 (0.874–1.255)	0.618		
T2DM (yes/no)	1.267 (1.005–1.598)	0.045*	1.067 (0.843–1.349)	0.591
Tumor site (colon/ rectum)	1.160 (0.990–1.359)	0.067		
Tumor stage (III/II/I)	2.073 (1.831–2.346)	0.000*	2.098 (1.853–2.377)	0.000*
Smoking (yes/no)	1.075 (0.914–1.264)	0.382		
Drinking (yes/no)	1.040 (0.876–1.234)	0.654		
IBL (larger/smaller)	1.465 (1.242–1.727)	0.000*	1.352 (1.142–1.601)	0.000*
IBT (yes/no)	2.107 (1.442–3.080)	0.000*	1.487 (1.011–2.188)	0.044*
Overall complications (yes/no)	1.817 (1.539–2.146)	0.000*	1.439 (1.201–1.723)	0.000*
Major complications (yes/no)	2.991 (2.154–4.154)	0.000*	2.431 (1.708–3.459)	0.000*

*P < 0.05.

*HR, hazard ratio; CI, confidence interval; BMI, body mass index; T2DM, type 2 diabetes mellitus; IBL, intraoperative blood loss; IBT, intraoperative blood transfusion*.

In terms of DFS, older age (*p* = 0.000, HR = 1.718, 95% CI = 1.480–1.994), advanced tumor stage (*p* = 0.000, HR = 2.061, 95% CI = 1.841–2.307), overall complications (*p* = 0.001, HR = 1.344, 95% CI = 1.137–1.588), larger IBL (*p* = 0.000, HR = 1.338, 95% CI = 1.150–1.558), and major complications (*p* = 0.000, HR = 2.187, 95% CI = 1.549–3.087) were independent predictors. However, IBT (*p* = 0.179, HR = 1.300, 95% CI = 0.886–1.908) was not an independent predictor of DFS (Table 8).

**Table 8:** Univariate and multivariate analysis of disease-free survival.

**Risk factors**	**Univariate analysis**		**Multivariate analysis**	
	**HR (95% CI)**	***P*-value**	**HR (95% CI)**	***P*-value**
Age (>/ ≤ 64, years)	1.852 (1.598–2.146)	0.000*	1.718 (1.480–1.994)	0.000*
Sex (female/male)	0.902 (0.778–1.046)	0.172		
BMI (>/ ≤ 22.6)	0.854 (0.739–0.988)	0.034*	0.930 (0.804–1.077)	0.332
Hypertension (yes/no)	1.038 (0.880–1.224)	0.659		
T2DM (yes/no)	1.136 (0.914–1.412)	0.252		
Tumor site (colon/ rectum)	1.085 (0.939–1.254)	0.268		
Tumor stage (III/II/I)	2.039 (1.823–2.282)	0.000*	2.061 (1.841–2.307)	0.000*
Smoking (yes/no)	1.085 (0.936–1.257)	0.279		
Drinking (yes/no)	1.036 (0.886–1.211)	0.661		
IBL (larger/smaller)	1.415 (1.220–1.642)	0.000*	1.338 (1.150–1.558)	0.000*
IBT (yes/no)	1.802 (1.235–2.630)	0.002*	1.300 (0.886–1.908)	0.179
Overall complications (yes/no)	1.647 (1.411–1.923)	0.000*	1.344 (1.137–1.588)	0.001*
Major complications (yes/no)	2.561 (1.855–3.536)	0.000*	2.187 (1.549–3.087)	0.000*

*P < 0.05.

*HR, hazard ratio; CI, confidence interval; BMI, body mass index; T2DM, type 2 diabetes mellitus; IBL, intraoperative blood loss; IBT, intraoperative blood transfusion*.

**Figure 2:**
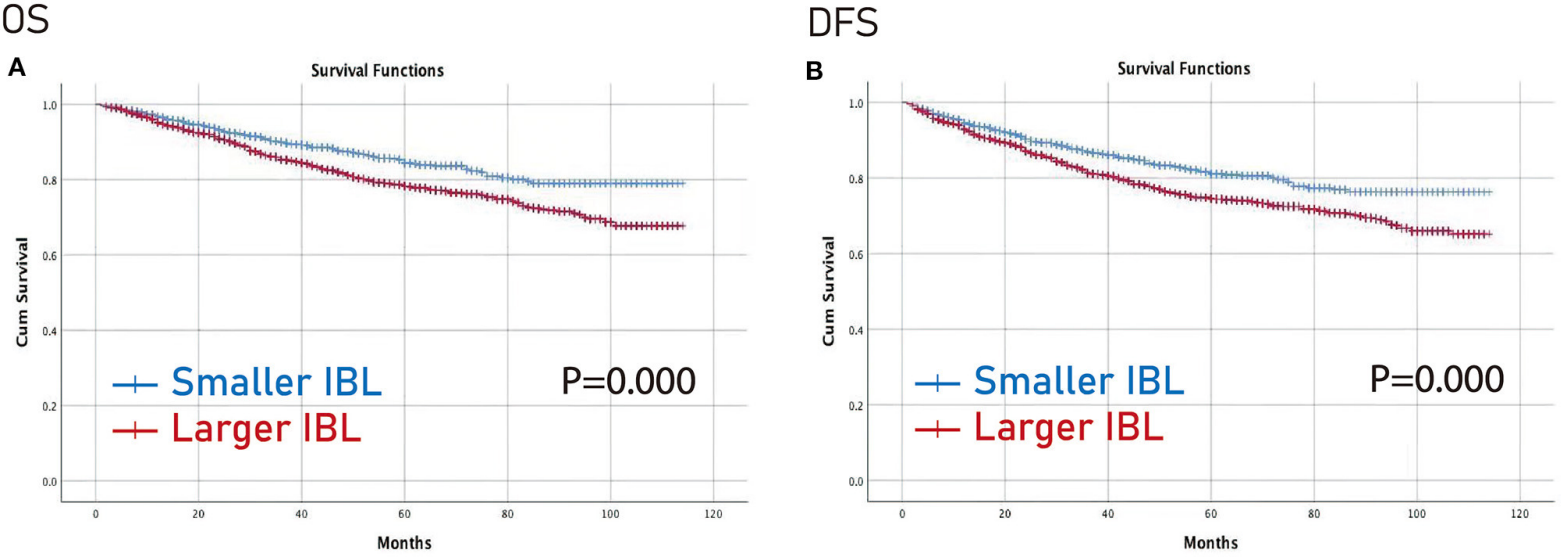
Prognosis of IBL on patients with CRC. **(A)** OS; **(B)** DFS. OS, overall survival; DFS, disease-free survival; IBL, intraoperative blood loss; CRC, colorectal cancer.

**Figure 3:**
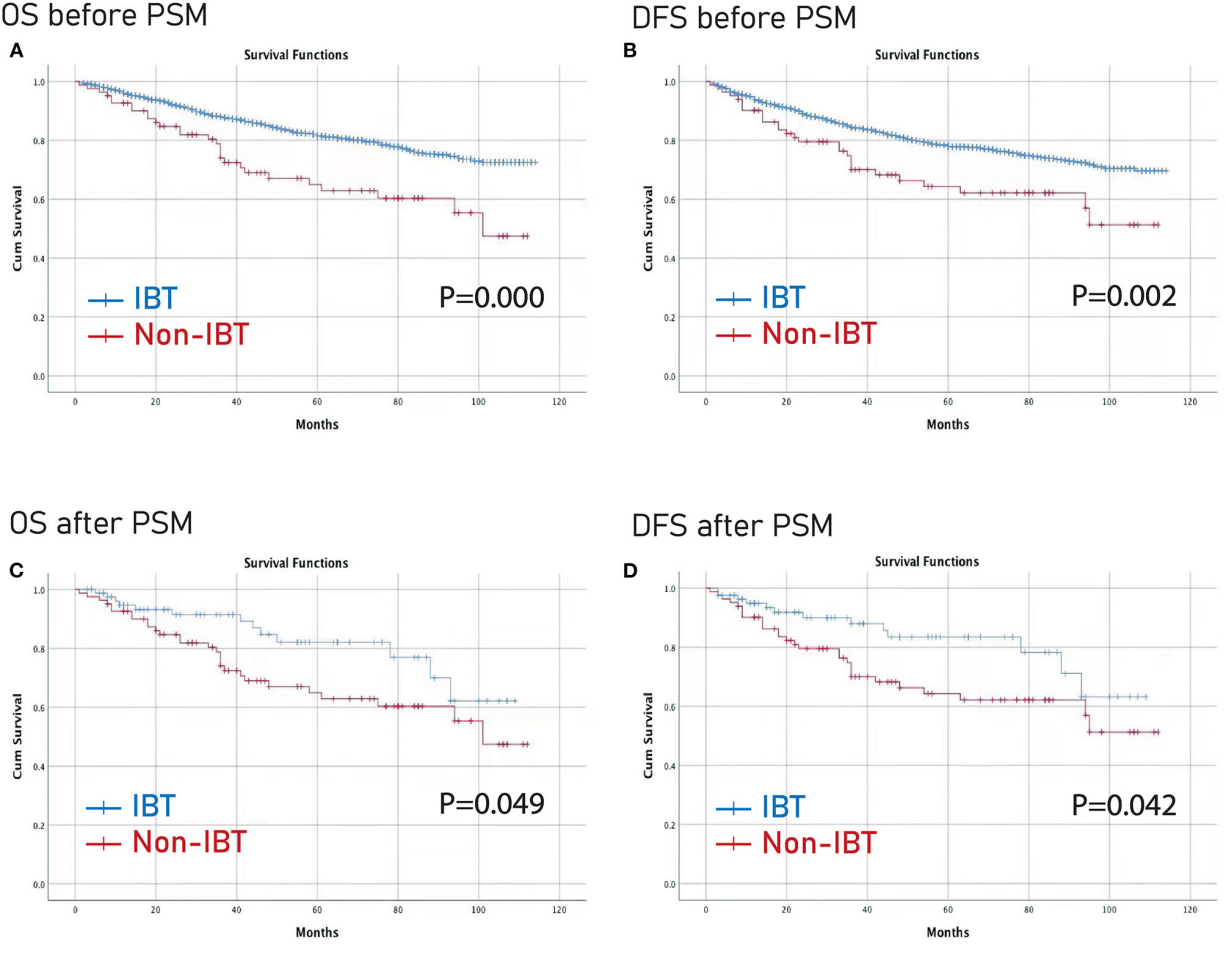
Prognosis of IBT on patients with CRC before and after PSM. **(A)** OS before PSM; **(B)** DFS before PSM; **(C)** OS after PSM; **(D)** DFS before PSM. OS, overall survival; DFS, disease-free survival; PSM, propensity score matching; IBL, intraoperative blood transfusion; CRC, colorectal cancer.

Furthermore, the Kaplan–Meier curve was conducted to compare the difference between the larger IBL group and the smaller IBL group, and between the IBT group and the non-IBT group. The larger IBL group had worse OS and DFS than the smaller IBL group (*p* < 0.01) (Figure 2). Moreover, the IBT group had worse OS and DFS before and after PSM than the non-IBT group (*p* < 0.05) (Figure 3).

## Discussion

In this study, there were 1,911 patients in the larger IBL group and 2,339 patients in the smaller IBL group. As for IBT, after 1:1 ratio PSM, there were 82 patients in the IBT group and 82 patients in the non-IBT group. The larger IBL group had longer operation time, longer post-operative hospital stay, smaller retrieved lymph nodes, and higher overall complication than the smaller IBL group. The IBT group had longer operation time, longer hospital stays, and higher overall complications compared with the non-IBT group in terms of short-term outcomes. Larger IBL and IBT were independent predictive factors of OS.

The amount of IBL could reflect the difficulty of surgery. There was a controversy of prognosis regarding IBL for patients with CRC (16–20, 25–27). Egenvall et al. reported that larger IBL increases the risk of later surgery for small bowel obstruction caused by tumor recurrence and surgical complications, but OS was not affected (20). Another study also reported IBL was not associated with increased median length of hospital stay nor did it increase the 30-day readmission rate (19). However, other studies reported that larger IBL was associated with increased complications or poor prognosis (16–18). Therefore, it was necessary to analyze the effect of IBL on the short-term outcomes and prognosis of patients who underwent primary CRC surgery.

However, the cut-off value of IBL was inconsistent in previous studies. We concluded the baseline information, cut-off value of IBL, and outcomes of previous studies in Table 9. The cut-off value of IBL was 50 ml, 100 ml, 200 ml, 250 ml, 450 ml, 800 ml, and 1,400 ml (16–20, 25–27). In this study, the cut-off value of IBL was the 75th percentile of IBL (100 ml), which was according to a previous study (16).

**Table 9:** Previous studies reporting the IBL on the outcomes of CRC patients.

**References**	**Country**	**Sample size**	**Cut-off IBL**	**Patients**	**Outcomes**
Okamura (16)	Japan	1,554	200 ml	Stage I/III CRC	IBL is associated with postoperative morbidity and survival in very elderly CRC patients.
Tamagawa (17)	Japan	1,597	200 ml	Stage II/III CRC	IBL was associated with significant differences in the OS and DFS of patients with stage II/III CRC patients.
Jiang (18)	China	139	250 ml	CRLM	IBL during CRLM resection is an independent predictor of long term survival and tumor recurrence.
Saleh (19)	United Kingdom	65	50 ml, 150 ml	CRC	IBL was not associated with increased median length of stay nor did it increase the 30 day re-admission rate.
Egenvall (20)	Sweden	1,843	450 ml, 800 ml, 1400 ml	RC	Major blood loss during surgery for RC increases the risk of later surgery for small bowel obstruction caused by tumor recurrence and surgical complications, but overall survival is not affected.
Shibutani (25)	Japan	277	100 ml	Stage II/III CRC	IBL was associated with poor long-term survival.
Mörner (26)	Sweden	3,554	250 ml	CC	IBL during surgery for colon cancer is an independent risk factor for later surgery for small bowel obstruction caused by tumor recurrence.
Mörner (27)	Sweden	3,062	250 ml	CC	IBL during surgery for colon cancer is a factor that influences long-term survival.

*CRC, colorectal cancer; IBL, intraoperative blood loss; CC, colon cancer; OS, overall survival; DFS, disease-free survival; CRLM, colorectal cancer liver metastasis; RC, rectal cancer*.

Perioperative blood transfusion might affect the complications, hospital stay, short-term death, and prognosis (11–15). However, no previous studies analyzed the effect of IBT on the short-term outcomes or prognosis. In this study, we analyzed IBT on the outcomes of CRC surgery; furthermore, PSM was conducted to minimize the bias of baseline information and the results would be more robust after PSM.

In terms of short-term outcomes, we found that larger IBL and IBT prolonged operation time and hospital stay, and increased complications. The prolonged hospital stay might be due to post-operative complications, and the recovery of intestinal peristalsis might be affected by larger IBL and IBT, which resulted in prolonged hospital stay. Therefore, surgeons should try to ensure the accuracy of the operation to avoid larger IBL or IBT.

In this study, larger IBL was an independent predictive factor of OS and DFS. The mechanism might be as follows: the animal experiments show that the activity or cytotoxicity of natural killer cells was reduced after blood loss, and the degree of reduction was related to the amount of blood loss (28, 29). IBL might increase the risk of tumor spread through blood during CRC surgery, which might lead to early recurrence and poor prognosis (30). In addition, larger IBL could lead to insufficient tissue perfusion and insufficient oxygenation (18). These events inhibited mitogen-induced lymphocyte proliferation and the production of interleukin-2 (31, 32), thus hindering anti-tumor immunity and upregulating vascular endothelial growth factor, which accelerated tumor angiogenesis (33, 34). Moreover, hypoxia could promote genome instability and lead to various genetic changes, which resulted in more aggressive phenotypes of residual tumor cells (35).

Intraoperative blood transfusion was also an independent predictive factor of OS. The potential hypothesis was that IBT might cause immune disturbances through transfused leukocytes, including changes in circulating lymphocytes, B cell function, suppressor T cell ratios, and helper T cells (12, 36). Another possible interpretation was that the need for IBT was a marker for sicker patients who were more likely to have worse prognosis than non-IBT patients (37).

There were some limitations in this study. First, this was a single-center retrospective study, which might cause selection bias; second, the follow-up time was relatively short and long-term follow-up is needed in the future; third, the information of classification and differentiation of tumors was lacking; fourth, the preoperative hemoglobin, the kind and volume of transfusion were lacking as well. Therefore, larger sample size with detailed information and long-term follow-up should be conducted in the following experiments.

In conclusion, larger IBL and IBT were associated with longer operation time, longer hospital stay, higher overall complications, and poorer OS. Based on the short-term outcomes and prognosis of IBL and IBT, surgeons should be cautious and more careful during the operation, and they require proficient surgical skills.

## Data Availability Statement

The datasets used and analyzed during the current study are available from the corresponding author on reasonable request.

## Ethics Statement

The studies involving human participants were reviewed and approved by the Ethics Committee of the First Affiliated Hospital of Chongqing Medical University. The patients/participants provided their written informed consent to participate in this study.

## Author Contributions

DP and BK contributed to the conception and design of the study and wrote the first draft of the manuscript. BK organized the database. DP performed the statistical analysis. X-YL, Z-WL, CY, BZ, and Z-QW wrote sections of the manuscript. All authors contributed to manuscript revision, read, and approved the submitted version.

## Funding

This study was supported by Chongqing Key Diseases Research and Application Demonstration Program (Colorectal Cancer Prevention and Treatment Technology Research and Application Demonstration [No. 2019ZX003]).

## Conflict of Interest

The authors declare that the research was conducted in the absence of any commercial or financial relationships that could be construed as a potential conflict of interest.

## Publisher's Note

All claims expressed in this article are solely those of the authors and do not necessarily represent those of their affiliated organizations, or those of the publisher, the editors and the reviewers. Any product that may be evaluated in this article, or claim that may be made by its manufacturer, is not guaranteed or endorsed by the publisher.
